# Self-supervised 3D deep learning on preoperative contrast-enhanced computed tomography for predicting high pathologic nodal burden in esophageal squamous cell carcinoma: temporal and external multicohort validation

**DOI:** 10.3389/fmed.2026.1841260

**Published:** 2026-06-11

**Authors:** Qian Li, Yongxin Li, Jing Bai, Jinze Zhang, Zhenkai Nie, Yanxin Ren, Zhantao Li, Jin Guo, Meng Li, Tingting Zhang, Xinbo Liu

**Affiliations:** 1 Department of Thoracic Surgery, The Fourth Hospital of Hebei Medical University, Shijiazhuang, China; 2 Department of Thoracic Surgery, Beijing Genertec Aerospace Hospital, Beijing, China; 3 School of Automation and Intelligence, Beijing Jiaotong University, Beijing, China; 4 Department of Pharmacy, The Fourth Hospital of Hebei Medical University, Shijiazhuang, China; 5 School of Medical Technology, Beijing Institute of Technology, Beijing, China; 6 Department of Radiology, The Fourth Hospital of Hebei Medical University, Shijiazhuang, China; 7 Department of Radiology, Yichang Central People's Hospital, Yichang, China

**Keywords:** contrast-enhanced computed tomography, deep learning, esophageal squamous cell carcinoma, external validation, nodal staging, risk prediction, self-supervised learning

## Abstract

**Background:**

Accurate preoperative identification of high nodal burden (pathologic N2 or N3; hereafter N2+) is important in esophageal squamous cell carcinoma, but contrast-enhanced computed tomography criteria based mainly on nodal size and morphology have limited sensitivity.

**Methods:**

In this retrospective multicohort study, 1,060 consecutive patients with esophageal squamous cell carcinoma who underwent preoperative contrast-enhanced computed tomography and curative-intent esophagectomy with lymphadenectomy were enrolled from two centers. Center A contributed a development cohort (*n* = 612; train/validation/internal test, 428/92/92) and a temporally held-out cohort (*n* = 238), and Center B contributed an external test cohort (*n* = 210). A three-dimensional residual convolutional neural network encoder was pretrained on 3,200 unlabeled chest computed tomography examinations using masked-volume reconstruction and then fine-tuned on tumor-centered volumes comprising the primary tumor plus a 5-mm margin.

**Results:**

The self-supervised model achieved area under the receiver operating characteristic curve values of 0.881 (95% confidence interval, 0.793–0.955) in the internal test cohort, 0.860 (95% confidence interval, 0.810–0.903) in the temporal cohort, and 0.860 (95% confidence interval, 0.810–0.906) in the external cohort. In the external cohort, sensitivity and specificity at the main operating point were 0.581 and 0.845 for the self-supervised model versus 0.339 and 0.784 for the guideline-inspired comparator. Calibration also improved with self-supervised pretraining (Brier score, 0.148; expected calibration error, 0.053).

**Conclusion:**

A contrast-enhanced computed tomography-only self-supervised three-dimensional model predicted high pathologic nodal burden in esophageal squamous cell carcinoma with robust temporal and external validation and showed numerically higher performance than a transparent computed tomography-only comparator. Calibrated risk estimates may help prioritize additional nodal workup when staging resources are limited or routine computed tomography findings are equivocal.

## 1 Introduction

Accurate preoperative nodal staging remains central in esophageal squamous cell carcinoma (ESCC) because nodal burden influences multimodality therapy, lymphadenectomy, prognosis, and the urgency of additional staging. Current practice guidelines recommend contrast-enhanced CT as a foundational staging examination, complemented when feasible by endoscopic ultrasound (EUS) with or without fine-needle aspiration and/or FDG PET/CT before curative treatment is finalized (1–5). Within the AJCC 8th edition framework, pathologic nodal categories reflect the number of metastatic regional lymph nodes; N2 + (N2 or N3, ≥3 metastatic regional nodes) is especially relevant because it identifies a high-burden subgroup more likely to require treatment intensification (4). Because the AJCC pN categories are defined by the number of metastatic regional lymph nodes, N2–N3 disease represents a larger metastatic reservoir than N1 disease and is generally associated with more advanced anatomic stage, higher recurrence risk, and stronger consideration of neoadjuvant or intensified multimodality strategies in guideline-based care (1, 2, 4). Thus, N2 + was selected as the primary endpoint not to diminish the clinical relevance of N1 disease, but to focus on a high-burden subgroup in which preoperative under-recognition is most likely to alter staging workup and treatment planning.

CT-based nodal assessment is imperfect when it relies mainly on size and morphology. Reactive lymph nodes may be enlarged, whereas micrometastatic disease may occur in non-enlarged nodes, leading to substantial false-negative and false-positive overlap (3, 5). Recent ESCC radiomics and deep learning studies suggest that CT can encode additional information related to lymphatic spread, but many studies target any nodal metastasis rather than clinically consequential high nodal burden, use relatively small single-center datasets, or lack rigorous temporal and external validation (6–8). Generalization is further challenged by domain shift across scanners, protocols, and calendar periods (9).

Self-supervised learning (SSL) offers a practical way to exploit large unlabeled imaging archives and learn transferable volumetric representations before downstream fine-tuning on comparatively scarce pathology-labeled data (10–12). We therefore developed a CT-only self-supervised 3D model that predicts pathologic N2 + disease from tumor-centered preoperative CECT volumes. We evaluated discrimination, calibration, and clinical utility across internal, temporal, and external cohorts and benchmarked the model against guideline-inspired CT criteria and an otherwise identical 3D network trained from scratch.

## 2 Materials and methods

### 2.1 Study design, reporting, and ethics

This retrospective multicohort analysis was designed as an imaging-only prediction-model study at two tertiary centers. Reporting was aligned with CLAIM and its 2024 update, TRIPOD+AI, and PROBAST+AI (13–16). The study was conducted in accordance with the Declaration of Helsinki and was approved by the Ethics Committee of The Fourth Hospital of Hebei Medical University (No. 20260158). A waiver of written informed consent was granted because of the retrospective design and the use of de-identified data.

### 2.2 Patients, endpoint, and cohort construction

Eligible patients had histopathologically confirmed ESCC, curative-intent esophagectomy with regional lymphadenectomy, preoperative CECT performed within 30 days before surgery, and a complete surgical pathology report enabling AJCC 8th edition nodal categorization. Patients were excluded if CT was obtained after neoadjuvant therapy, if distant metastatic disease was present at diagnosis, if CT quality was non-diagnostic, or if the imaging series or pathology report was incomplete.

**Figure 1:**
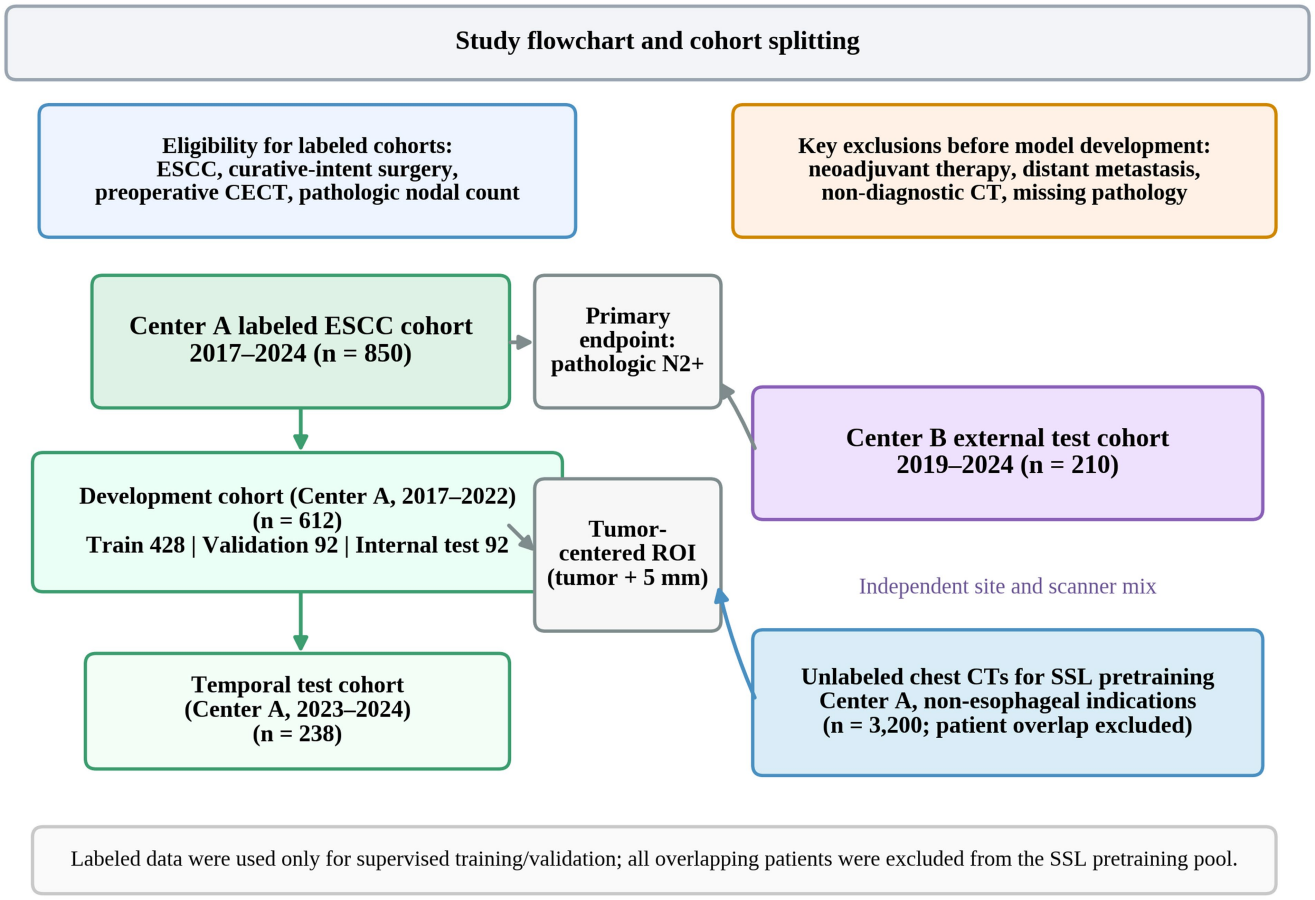
Study flowchart and cohort splitting. The diagram summarizes labeled cohort construction, temporal and external validation, and the unlabeled chest computed tomography pool used for self-supervised pretraining.

The labeled dataset comprised 1,060 non-overlapping patients (Figure 1). Center A contributed a development cohort of 612 consecutive patients scanned from January 2017 through December 2022; this cohort was split at the patient level into training (*n* = 428), validation (*n* = 92), and internal test (*n* = 92) subsets stratified by N2 + prevalence. A temporally held-out Center A cohort (January 2023 through June 2024) provided 238 patients for temporal validation. Center B contributed 210 consecutive patients (January 2019 through June 2024) for external validation.

The primary endpoint was pathologic N2 + disease, defined as pathologic N2 or N3 (≥3 metastatic regional lymph nodes) according to AJCC 8th edition criteria (4). Patients with 0–2 metastatic regional nodes were grouped as non-N2 + .

### 2.3 Image acquisition, segmentation, and tumor-centered volumes

All labeled cohorts used standard-of-care CECT. Although acquisition parameters varied across sites and scanners, typical examinations used 120 kVp, automated tube-current modulation, reconstructed slice thicknesses of 1.0–2.5 mm, and intravenous iodinated contrast at 1.5–2.0 mL/kg delivered at 3–4 mL/s. DICOM series were converted to NIfTI, resampled to isotropic 1.0-mm voxels, clipped to −150 to 250 Hounsfield units, and normalized to zero mean and unit variance on a per-volume basis.

Primary-tumor segmentation on the CECT series was independently performed by two radiologists (each with ≥5 years of experience) and subsequently reviewed by two thoracic surgeons (each with ≥5 years of experience), all blinded to final pathologic nodal status. Interobserver agreement was quantified using the Dice similarity coefficient in a randomly selected 60-case subset. A tumor-centered ROI was generated from the minimal 3D tumor bounding box expanded by an isotropic 5-mm margin to include peritumoral tissue; the final input crop was fixed at 128 × 128 × 96 voxels. Sensitivity analyses tested alternative margins of 0 mm and 10 mm.

### 2.4 Self-supervised pretraining and downstream model development

**Figure 2:**
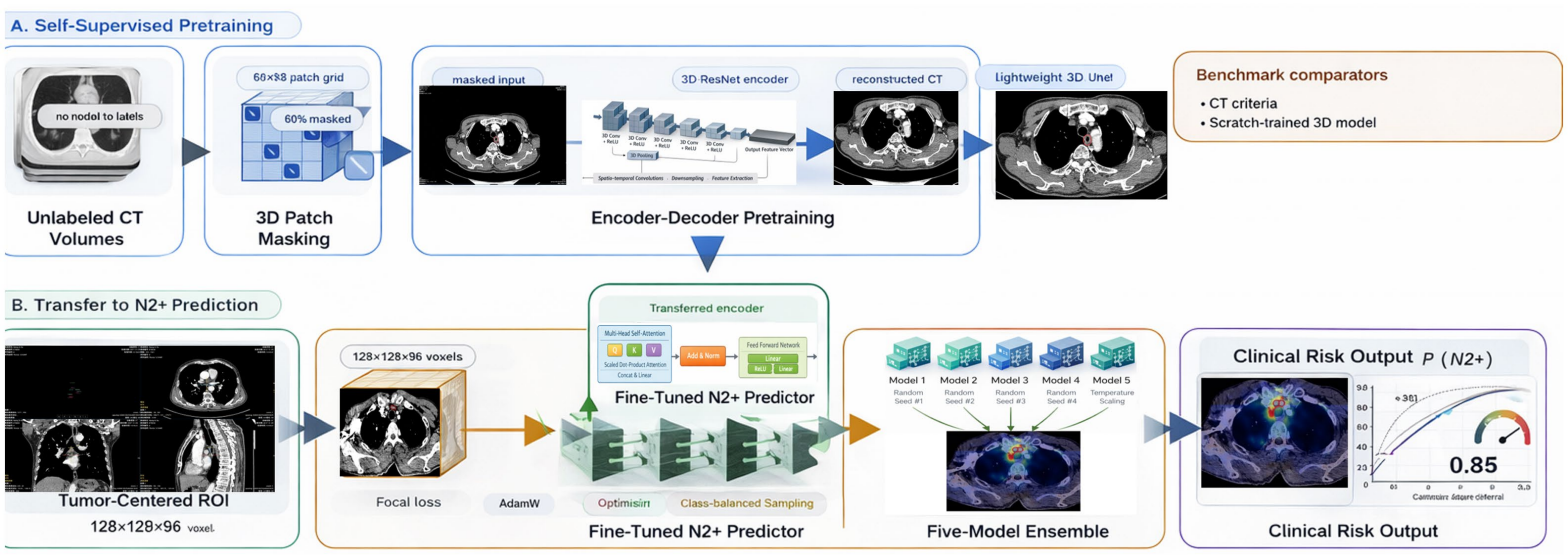
CT-only self-supervised modeling workflow. Self-supervised pretraining on unlabeled chest computed tomography volumes using masked patch reconstruction was followed by transfer learning and fine-tuning on tumor-centered volumes for prediction of high pathologic nodal burden. CT, Computed Tomography.

Figure 2 summarizes the full modeling workflow. The downstream classifier used a 3D residual convolutional neural network encoder adapted for volumetric CT and conceptually based on a ResNet-18-style backbone (17). The encoder used an initial 7 × 7 × 7 convolution and max-pooling layer, followed by four residual stages; each stage contained two basic residual blocks with 3 × 3 × 3 convolutions. Channel widths were 32, 64, 128, and 256, with stride-2 downsampling at the first block of stages 2–4. Group normalization (8 groups) and rectified linear-unit activations were used after convolutional layers. Global average pooling produced a 256-dimensional feature vector, which was passed to a two-layer multilayer perceptron head with 128 hidden units, dropout of 0.3, and a sigmoid output.

An end-to-end CNN design was chosen because the SSL reconstruction pretext task and downstream classifier could share the same encoder, allowing task-specific fine-tuning of volumetric tumor and peritumoral representations. This design was primarily motivated by compatibility with SSL pretraining and by the ability to learn task-specific 3D features without a separate hand-crafted feature-selection step. Radiomics and hybrid deep-feature pipelines remain valuable alternatives, but they introduce feature discretization, selection, and harmonization choices that can be difficult to standardize across centers.

For SSL pretraining, we assembled 3,200 unlabeled chest CT examinations from Center A obtained for non-esophageal indications, after excluding all patients who appeared in the labeled ESCC cohorts. The unlabeled pool included examinations performed for routine thoracic assessment, lung-nodule evaluation or surveillance, suspected inflammatory or infectious lung disease, chronic lung disease, and non-esophageal oncologic follow-up; no scan was selected according to ESCC status or nodal burden. These cases were used to learn CT-domain representations, including tissue-air interfaces, soft-tissue texture, spatial continuity, and scanner/protocol variation, rather than ESCC-specific nodal labels. A masked-volume reconstruction task was used, inspired by prior 3D medical SSL and masked autoencoding frameworks (10, 11). CT volumes were partitioned into non-overlapping 8 × 8 × 8 voxel patches, 60% of patches were randomly masked, and the encoder learned to reconstruct masked voxels through a lightweight decoder. Masking was performed by spatially random patch selection, and reconstruction loss was calculated as mean squared error over masked voxels only. The decoder consisted of two 3D transposed-convolution upsampling layers followed by a 1 × 1 × 1 convolution for voxel-intensity reconstruction. Pretraining used AdamW optimization (18) with an initial learning rate of 1 × 10^−4^, batch size of 8, 200 epochs, 10 warm-up epochs, cosine annealing, weight decay of 1 × 10^−2^, mixed precision, and on-the-fly augmentations including flips, mild rotations, Gaussian noise, and intensity scaling.

For downstream N2 + classification, the pretrained encoder was fine-tuned end-to-end using focal loss (19) with class-balanced sampling and early stopping based on validation AUC. Fine-tuning used AdamW with an initial learning rate of 3 × 10–5, batch size of 8, a maximum of 150 epochs, early-stopping patience of 20 epochs, focal-loss *γ* = 2.0, and class weights inversely proportional to class prevalence. A five-member deep ensemble was trained with different random seeds to stabilize predictions and quantify model uncertainty (20). Temperature scaling was then fitted on the validation set to improve probability calibration without altering rank order (21).

### 2.5 Comparator models and statistical evaluation

Two principal comparators were evaluated. First, a supervised-from-scratch model used the same 3D architecture and optimization strategy but random initialization instead of SSL pretraining. Second, a guideline-inspired CT comparator approximated routine CT nodal interpretation. Two thoracic radiologists, blinded to pathology, classified nodal stations as suspicious if a node had a short-axis diameter of at least 8–10 mm and/or malignant morphology such as roundness, central necrosis, or clustered nodal disease. CT-predicted N2 + was defined as at least three suspicious regional nodes or bulky multistation nodal disease; this rule was intentionally transparent and reproducible, but it was not an official guideline definition. The comparators were chosen to probe different questions: the guideline-inspired comparator represented direct nodal CT interpretation, whereas the from-scratch network isolated the effect of SSL initialization. Because the AI input was tumor-centered and the CT comparator was node-centered, the comparison was interpreted as testing complementary CT information rather than as replacing radiologic nodal assessment.

The prespecified primary performance metric was AUC in the external cohort. Secondary metrics included AUC in the internal and temporal cohorts; sensitivity, specificity, accuracy, positive predictive value (PPV), and negative predictive value (NPV) at the validation-set Youden threshold; Brier score and expected calibration error (ECE); and decision-curve analysis (DCA) using net benefit (22). Prespecified subgroup analyses in the external cohort were stratified by tumor location, slice thickness, and scanner vendor. Additional exploratory *post hoc* subgroup analyses were conducted for arterial- versus venous-phase examinations after excluding mixed/incomplete-protocol cases, clinical T category (T1-2 vs. T3-4), and maximum CT tumor diameter dichotomized at the external-cohort median. Sensitivity analyses examined alternative ROI definitions, test-time augmentation, segmentation perturbation, and exclusion of very small tumors. Gradient-weighted class activation mapping (Grad-CAM) was used for qualitative interpretability analysis (23). Continuous variables were summarized as mean ± standard deviation or median [interquartile range], and categorical variables as counts and percentages. Cross-cohort comparisons used one-way analysis of variance or Kruskal–Wallis tests for continuous variables and chi-square or Fisher exact tests for categorical variables, as appropriate. Ninety-five percent confidence intervals for AUCs were estimated with 2,000 patient-level bootstrap resamples stratified by outcome; exact binomial intervals were used for operating characteristics. Paired ROC comparisons with DeLong tests were performed as exploratory analyses when paired predictions were available. Because multiple pairwise comparisons were not adjusted for multiplicity, between-model differences were interpreted cautiously. Statistical analyses used Python 3.10 with PyTorch, scikit-learn, and statsmodels, and R version 4.3.2.

All models were implemented in PyTorch and trained on NVIDIA A100 GPUs using mixed precision. Hyperparameters were tuned only on the training and validation data; the internal, temporal, and external test cohorts were not used for model selection.

## 3 Results

### 3.1 Patient characteristics

Baseline characteristics and imaging summaries are presented in Table 1. Pathologic N2 + prevalence was 28.4% (174/612) in the Center A development cohort, 26.5% (63/238) in the temporal cohort, and 29.5% (62/210) in the external cohort. The external cohort also differed from Center A in scanner-vendor distribution, supporting the relevance of external generalization testing. Clinical T category and maximum CT tumor diameter were considered expected macroscopic correlates of nodal burden; therefore, model performance was interpreted cautiously, and the absence of a formal clinical-feature logistic-regression benchmark was treated as a limitation of incremental-value inference.

**Table 1:** Baseline characteristics and computed tomography acquisition summary.

Variable	Development (Center A, 2017–2022) (*n* = 612)	Temporal (Center A, 2023–2024) (*n* = 238)	External (Center B, 2019–2024) (*n* = 210)	*p* value
Age, years (mean ± SD)	62.1 ± 8.9	63.0 ± 8.4	61.5 ± 9.1	0.19
Sex, male (%)	459 (75.0)	180 (75.6)	160 (76.2)	0.94
Tumor location, upper/middle/lower	98/354/160	40/132/66	60/110/40	<0.001
Clinical T category, T1–2/T3–4	226/386	82/156	70/140	0.58
Pathologic N category, N0–1/N2–3	438/174	175/63	148/62	0.76
Slice thickness ≤1.5 mm (%)	210 (34.3)	85 (35.7)	60 (28.6)	0.22
Scanner vendor, Siemens/GE	350/262	140/98	90/120	<0.001
Contrast phase (arterial/venous/mixed)†	410/160/42	160/60/18	120/70/20	0.11
Time from CT to surgery, days (median [IQR])	12 [7–18]	11 [6–17]	13 [7–19]	0.27
Tumor longest diameter on CT, cm (median [IQR])	4.2 [3.2–5.4]	4.1 [3.0–5.3]	4.4 [3.3–5.6]	0.19

†SD, standard deviation; IQR, interquartile range. Mixed indicates variable phase timing or incomplete protocol documentation.

Interobserver segmentation agreement in the 60-case subset was high, with a median Dice similarity coefficient of 0.86 (interquartile range, 0.82–0.90). Modest segmentation perturbations did not materially change discrimination in sensitivity analysis, suggesting that the final model did not overfit to pixel-precise contour boundaries.

### 3.2 Primary model performance

**Figure 3:**
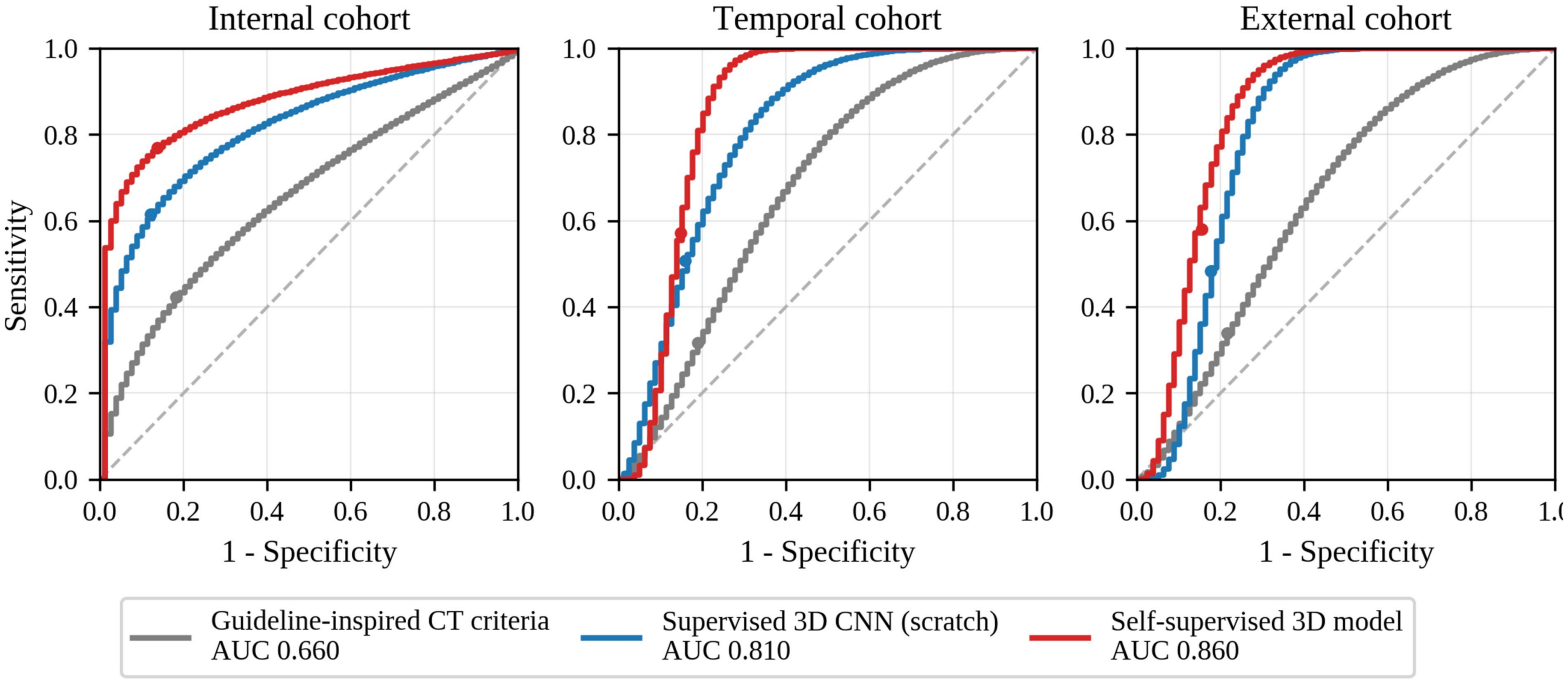
Receiver operating characteristic curves across the internal, temporal, and external cohorts. The self-supervised 3D model showed numerically higher AUCs than the guideline-inspired CT comparator and the supervised-from-scratch 3D baseline. Displayed AUC values correspond to Table 2. AUC, area under the receiver operating characteristic curve; CT, computed tomography. Axes and legends are harmonized across cohort panels.

**Figure 4:**
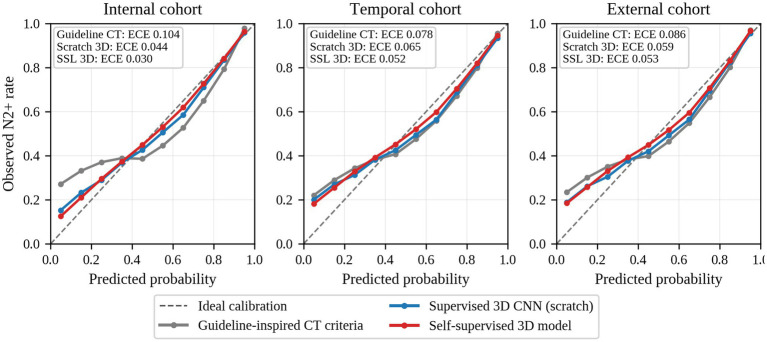
Calibration plots across the internal, temporal, and external cohorts after temperature scaling. The self-supervised 3D model remained closest to the identity line across cohorts, consistent with the lower expected calibration error values reported in Table 2. ECE, expected calibration error. Axes and legend formatting are standardized across panels.

Table 2 and Figure 3 summarize discrimination, operating characteristics, and probability quality across cohorts. The self-supervised model achieved AUCs of 0.881 (95% CI: 0.793–0.955) in the internal test cohort, 0.860 (0.810–0.903) in the temporal cohort, and 0.860 (0.810–0.906) in the external cohort. Across all three cohorts, the self-supervised model showed numerically higher AUCs than both the guideline-inspired CT comparator and the same 3D architecture trained from scratch; however, between-model comparisons were interpreted cautiously because formal pairwise testing was exploratory and not adjusted for multiplicity. Calibration plots are shown in Figure 4; consistent with the lower Brier scores and ECE values in Table 2, the self-supervised model remained closest to the identity line after temperature scaling, particularly in the temporal and external cohorts.

**Table 2:** Performance across cohorts.

Model	Cohort	AUC (95% CI)	Sens	Spec	Acc	PPV	NPV	Brier	ECE
Guideline CT criteria	Internal test (*n* = 92)	0.660 (0.565–0.754)	0.423	0.818	0.707	0.478	0.783	0.194	0.104
Supervised 3D CNN (from scratch)	Internal test (*n* = 92)	0.821 (0.727–0.907)	0.615	0.879	0.804	0.667	0.853	0.144	0.044
Self-supervised 3D model (tumor + 5-mm margin)	Internal test (*n* = 92)	0.881 (0.793–0.955)	0.769	0.864	0.837	0.690	0.905	0.118	0.030
Guideline CT criteria	Temporal test (*n* = 238)	0.680 (0.610–0.744)	0.317	0.811	0.681	0.377	0.768	0.194	0.078
Supervised 3D CNN (from scratch)	Temporal test (*n* = 238)	0.810 (0.748–0.864)	0.508	0.840	0.752	0.533	0.826	0.156	0.065
Self-supervised 3D model (tumor + 5-mm margin)	Temporal test (*n* = 238)	0.860 (0.810–0.903)	0.571	0.851	0.777	0.581	0.847	0.138	0.052
Guideline CT criteria	External test (*n* = 210)	0.660 (0.590–0.728)	0.339	0.784	0.652	0.396	0.739	0.210	0.086
Supervised 3D CNN (from scratch)	External test (*n* = 210)	0.810 (0.742–0.866)	0.484	0.824	0.724	0.536	0.792	0.174	0.059
Self-supervised 3D model (tumor + 5-mm margin)	External test (*n* = 210)	0.860 (0.810–0.906)	0.581	0.845	0.767	0.610	0.828	0.148	0.053

AUC, area under the receiver operating characteristic curve; CI, confidence interval; Sens, sensitivity; Spec, specificity; Acc, accuracy; PPV, positive predictive value; NPV, negative predictive value; ECE, expected calibration error.

### 3.3 Clinical utility and risk stratification

At a higher-sensitivity triage threshold selected on the validation set (0.314), external-cohort sensitivity increased to 0.694 while specificity remained 0.757 (Table 3), supporting a workflow in which higher-risk patients are prioritized for additional nodal workup rather than directly upstaged by CT alone. Patients flagged at this threshold would be candidates for confirmatory evaluation, including multidisciplinary review, focused radiologic reassessment of regional nodal stations, FDG PET/CT when available, and EUS-guided fine-needle aspiration or other selective nodal sampling when feasible; the model output was not intended to directly upstage patients or determine treatment by itself.

**Table 3:** Triage operating point and risk thresholds in the external cohort.

Setting	Threshold	Sensitivity	Specificity	PPV	NPV	Flagged (%)
Youden (main analysis)	0.402	0.581	0.845	0.610	0.828	59/210 (28.1)
High sensitivity (triage)	0.314	0.694	0.757	0.544	0.855	79/210 (37.6)

**Figure 5:**
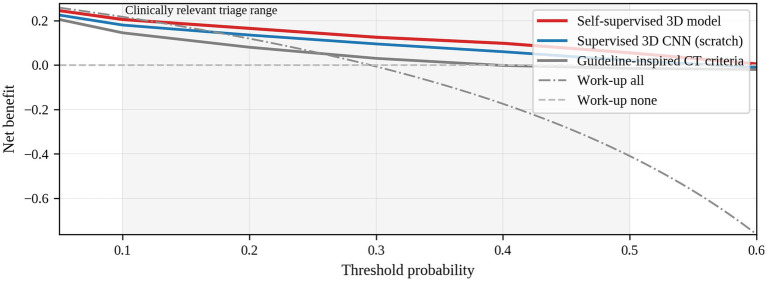
Decision curve analysis in the external cohort. Across clinically relevant threshold probabilities, the self-supervised 3D model showed higher net benefit than the guideline-inspired CT comparator and the supervised-from-scratch baseline in this validation cohort. Decision curves are interpreted as prioritization for additional nodal work-up rather than direct treatment decisions. Legends and axes are consistently formatted.

Risk groups were defined before external evaluation using fixed predicted-probability thresholds of <0.20 for low risk, 0.20–0.50 for intermediate risk, and >0.50 for high risk; these cut points were selected for clinical interpretability rather than optimized on the external cohort. Risk stratification was monotonic (Table 4). In the external cohort, observed N2 + prevalence was 11.1% in the low-risk group, 32.5% in the intermediate-risk group, and 65.0% in the high-risk group. Decision-curve analysis (Figure 5) also favored the self-supervised model across clinically relevant thresholds, consistent with a role in triage and risk enrichment rather than stand-alone treatment selection.

**Table 4:** Risk stratification by predicted probability in the external cohort.

Risk group	Probability range	Patients (n)	N2 + cases (n)	Observed N2 + prevalence
Low	<0.20	90	10	11.1%
Intermediate	0.20–0.50	80	26	32.5%
High	>0.50	40	26	65.0%

### 3.4 Ablation, uncertainty, subgroup analysis, and error patterns

**Figure 6:**
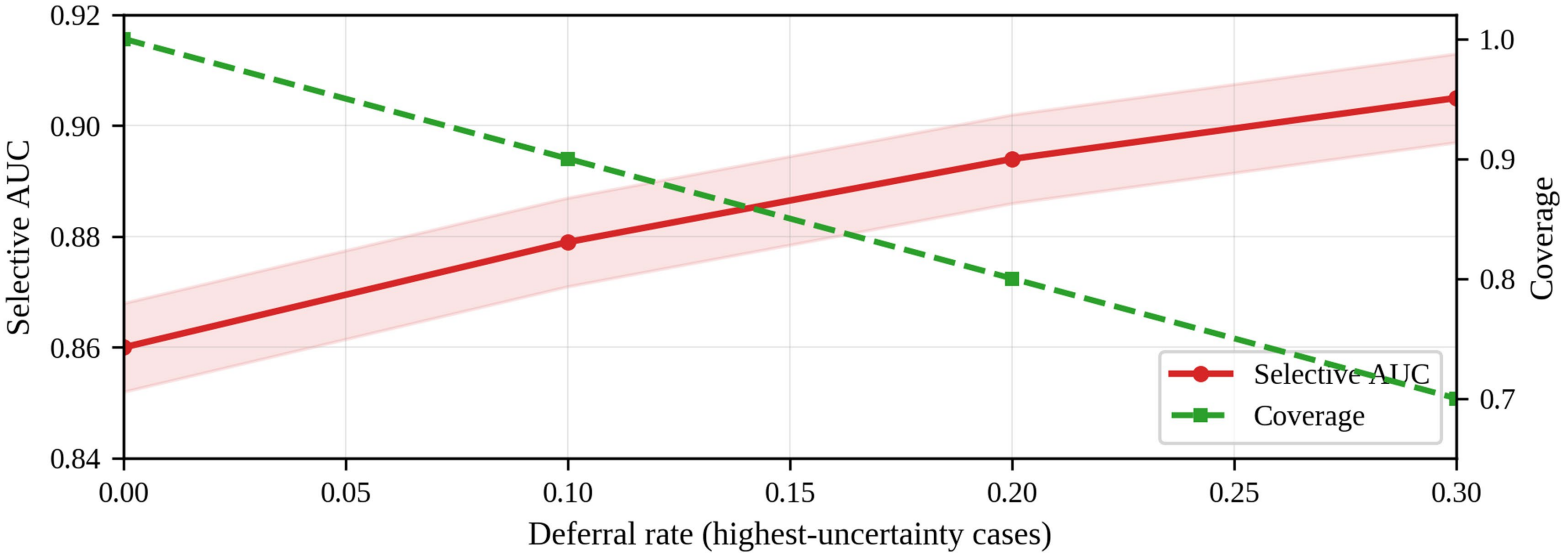
Uncertainty-aware selective prediction in the external cohort. Selective AUC increased as the most uncertain cases were deferred, while coverage decreased as expected, supporting a conservative workflow in which equivocal predictions trigger further staging rather than automatic classification. AUC, area under the receiver operating characteristic curve.

Uncertainty-aware selective prediction (Figure 6) showed that selective AUC in the external cohort increased from 0.860 at full coverage to approximately 0.905 after deferring the 30% most uncertain cases, supporting a conservative workflow in which equivocal predictions trigger additional staging rather than automatic classification. Ablation results in Table 5 show that both volumetric context and SSL pretraining contributed to performance. Relative to the final model, AUC fell to 0.821 when the 3D network was trained from scratch, to 0.846 when only tumor voxels were used, and to 0.790 when only peritumoral tissue was used. A 2D slice-based baseline also underperformed the final 3D approach. These ablations suggest that the model was not relying on the segmented tumor mass alone: tumor voxels provided intralesional signal, peritumoral tissue provided contextual information, and their combination was strongest when initialized with SSL.

**Table 5:** Ablation analysis in the internal test cohort.

Model variant	AUC (95% CI)	Brier	ECE
2D slice-based baseline	0.740 (0.620–0.840)	0.168	0.072
Supervised 3D CNN (from scratch; tumor + 5 mm)	0.821 (0.727–0.907)	0.144	0.044
Tumor-only ROI + SSL	0.846 (0.756–0.923)	0.131	0.048
Peritumoral-only ROI + SSL	0.790 (0.690–0.880)	0.154	0.060
Tumor + 5-mm margin ROI + SSL (final model)	0.881 (0.793–0.955)	0.118	0.030
Tumor + 5-mm margin ROI + SSL + test-time augmentation	0.892 (0.813–0.960)	0.114	0.028

ROI, region of interest; SSL, self-supervised learning.

**Figure 7:**
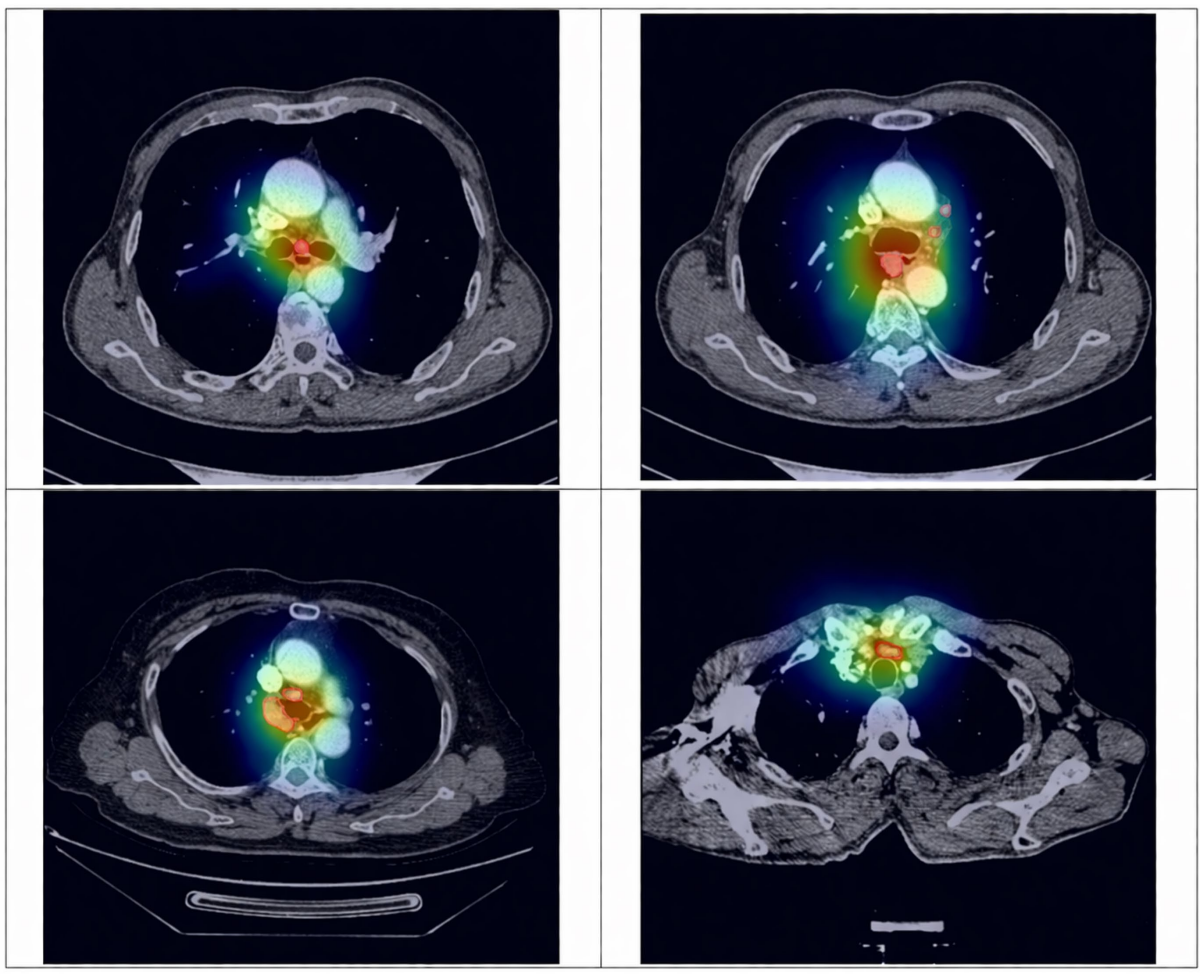
Representative Grad-CAM overlays from the external cohort. Heatmaps are concentrated around the tumor boundary and adjacent peritumoral tissue, supporting the hypothesis that the model captures interface-related cues associated with lymphatic spread. Grad-CAM, gradient-weighted class activation mapping.

External-cohort subgroup analyses were expanded to include tumor location, slice thickness, scanner vendor, contrast phase, clinical T category, and CT tumor-diameter strata (Table 6). The added phase analysis excludes mixed/incomplete-protocol cases, and the tumor-diameter strata are dichotomized at the external-cohort median. Grad-CAM visualizations (Figure 7) showed that the network concentrated attention at the tumor interface and adjacent soft tissues rather than on a single intratumoral hotspot alone. Confusion matrices in Table 7 show that, relative to guideline-inspired CT criteria, the self-supervised model reduced false negatives from 41 to 26 while also reducing false positives from 32 to 23. Final interpretation of the new subgroup estimates should remain cautious because each subgroup has fewer outcome events than the full external cohort.

**Table 6:** External-cohort subgroup and exploratory post hoc robustness analyses of the self-supervised model.

Subgroup	*n*	AUC (95% CI)
Tumor location: upper	60	0.855 (0.760–0.930)
Tumor location: middle	110	0.864 (0.800–0.920)
Tumor location: lower	40	0.848 (0.740–0.940)
Slice thickness ≤1.5 mm	60	0.872 (0.790–0.940)
Slice thickness >1.5 mm	150	0.834 (0.780–0.885)
Scanner: Siemens	90	0.858 (0.790–0.920)
Scanner: GE	120	0.862 (0.800–0.920)
Contrast phase: arterial (mixed/incomplete excluded)	120	0.862 (0.800–0.918)
Contrast phase: venous (mixed/incomplete excluded)	70	0.854 (0.762–0.928)
Clinical T category: T1-2	70	0.849 (0.755–0.925)
Clinical T category: T3-4	140	0.864 (0.804–0.916)
CT tumor diameter ≤ external-cohort median (4.4 cm)	105	0.851 (0.779–0.915)
CT tumor diameter > external-cohort median (4.4 cm)	105	0.868 (0.802–0.926)

**Table 7:** External-cohort confusion matrices (*n* = 210).

Method	TP	FP	TN	FN	Interpretation
Guideline CT criteria	21	32	116	41	High specificity but low sensitivity for N2+
Self-supervised 3D model	36	23	125	26	Improved balance of sensitivity, PPV, and NPV
High-sensitivity triage (SSL)	43	36	112	19	Prioritizes sensitivity at the cost of more workups

TP, true positive; FP, false positive; TN, true negative; FN, false negative; SSL, self-supervised learning.

## 4 Discussion

In this multicohort study, a CT-only self-supervised 3D model predicted pathologic N2 + nodal burden in ESCC with robust internal, temporal, and external validation. The model consistently showed numerically higher discrimination than a guideline-inspired CT-only comparator and the same 3D architecture trained from scratch, while formal superiority claims should be interpreted cautiously because pairwise comparisons were exploratory. The advantage was not limited to rank-order discrimination: probability quality and triage-oriented operating characteristics also improved after SSL pretraining.

The emphasis on N2 + rather than any nodal metastasis is clinically important. High nodal burden is more tightly linked to treatment escalation, unexpected postoperative upstaging, and adverse prognosis than single-node involvement alone (4). In contemporary practice, CT is usually the first staging examination, whereas EUS-FNA and PET/CT may be limited by access, scheduling, cost, or local expertise (1–5). A calibrated CT-only risk score could therefore function as an adjunctive triage tool, enriching for patients who should undergo expedited EUS-FNA, PET/CT, or more intensive nodal sampling rather than replacing guideline-based multimodality staging. The comparison with nodal CT criteria was intentionally asymmetric but clinically complementary: the AI model interrogated the primary tumor and immediately adjacent peritumoral tissue, whereas radiologists evaluated regional nodal size and morphology. Primary tumor/peritumoral features may capture surrogates of invasive growth, lymphovascular invasion, and local microenvironmental response even when metastatic nodes are not enlarged, while nodal morphology provides direct anatomic evidence when nodal disease is macroscopic.

Prior ESCC radiomics studies have shown that primary-tumor CT features can carry information about lymphatic dissemination, but many reports focused on any nodal metastasis, used single-center designs, or lacked robust external validation (6–8). Our ablation analyses suggest that two ingredients were especially important here: volumetric 3D context and self-supervised initialization. The complementary contribution of tumor and peritumoral voxels is biologically plausible, because the tumor boundary and adjacent soft tissue may reflect invasive growth, lymphovascular invasion surrogates, and local microenvironmental response. Subgroup analyses stratified by clinical T category and CT-measured tumor diameter were further conducted to assess whether the model primarily captured gross tumor size or T-stage-related information. These analyses should be regarded as sensitivity checks supporting the robustness of the mechanistic interpretation. The benefit of SSL is also concordant with broader medical-imaging literature showing that unlabeled pretraining can improve transferability when pathology-labeled datasets are modest in size (10–12). The selected 3D residual CNN was a pragmatic choice rather than an assertion that this backbone is universally optimal. Residual connections support stable optimization in moderately deep volumetric networks, 3D convolutions preserve craniocaudal and circumferential context that may be lost in 2D sampling, and the compact architecture was feasible for the available labeled dataset and ensemble training. Conceptually, the SSL stage is a form of within-modality transfer learning, but it differs from supervised ImageNet-style or disease-label pretraining because it uses unlabeled CT volumes and a reconstruction objective to learn general volumetric CT structure before pathology-specific fine-tuning. The heterogeneous non-esophageal pretraining pool could introduce representation bias if certain disease backgrounds dominate the learned features. We attempted to mitigate this risk by excluding ESCC cases and all overlapping patients, using a label-agnostic reconstruction objective, applying augmentation, and testing temporal and external cohorts; nevertheless, site-balanced and indication-stratified pretraining should be evaluated in future work.

Calibration mattered in this study because staging decisions are threshold-based. A model with acceptable discrimination but unreliable probability estimates can misstate how aggressively clinicians should pursue additional staging. The lower Brier scores and ECE values observed after SSL pretraining, together with the more favorable decision-curve and selective-prediction results (Figures 4–6), therefore add practical value beyond AUC alone. These properties support the use of the model as a risk-enrichment tool within a guideline framework rather than as an autonomous diagnostic or treatment-decision system (21, 22). Operationally, a high-sensitivity flag should trigger confirmatory staging rather than automatic treatment escalation, such as expert CT review, PET/CT when available, EUS-FNA of suspicious or accessible nodes, and multidisciplinary discussion of neoadjuvant-treatment planning.

This study has limitations. First, it was retrospective and limited to surgically treated patients with available pathology, which may not represent the full ESCC population, particularly patients managed non-surgically. This selection bias may limit calibration in patients treated with definitive chemoradiotherapy or best supportive care. Second, the workflow still required tumor segmentation, although interobserver agreement was high and automated segmentation could be incorporated in future iterations (24). Third, the model was deliberately CT-only to maximize portability, but multimodal approaches incorporating PET, EUS, and clinical biomarkers may achieve higher performance. Finally, the endpoint was patient-level high nodal burden rather than nodal-station localization; future work should test whether tumor-centered and node-centered representations provide complementary information and should prospectively monitor calibration under real-world dataset shift (9). Another limitation is the absence of a formal multivariable clinical-feature logistic-regression comparator incorporating clinical T category and maximum CT tumor diameter; therefore, the current results support improvement over CT nodal criteria and random initialization, but they do not by themselves prove absolute incremental value over every routine clinical model. External heterogeneity was useful for testing transportability, but differences in scanner vendor, slice thickness, and contrast phase may still affect calibration. Although an exploratory arterial-versus-venous phase analysis excluding mixed/incomplete-protocol cases has been performed, the resulting subgroup estimates should be considered *post hoc* and should be confirmed in prospective phase-stratified validation together with site-specific calibration monitoring.

Overall, these results support a pragmatic CT-first AI-assist strategy: conventional CECT remains the backbone of staging, while a calibrated model-generated N2 + risk score helps identify which patients most urgently need confirmatory nodal assessment. Prospective multicenter validation and clinical impact analysis are the next necessary steps before deployment. In such a workflow, low-risk predictions may support routine staging pathways, whereas high-risk or high-uncertainty predictions should prioritize confirmatory nodal assessment rather than replace guideline-based staging.

### 4.1 Conclusion

In patients with ESCC undergoing preoperative contrast-enhanced CT, a CT-only self-supervised 3D model predicted pathologic N2 + nodal burden with numerically higher discrimination, improved calibration, and greater triage-oriented utility than guideline-inspired CT criteria in this multicohort validation setting. These findings support further prospective testing of AI-enhanced CT as an adjunct to, not a replacement for, guideline-based staging.

## Data Availability

The raw data supporting the conclusions of this article will be made available by the authors, without undue reservation.
